# Universal Pacemaker of Genome Evolution

**DOI:** 10.1371/journal.pcbi.1002785

**Published:** 2012-11-29

**Authors:** Sagi Snir, Yuri I. Wolf, Eugene V. Koonin

**Affiliations:** 1 Department of Evolutionary and Environmental Biology and The Institute of Evolution, University of Haifa Mount Carmel, Haifa, Israel; 2 National Center for Biotechnology Information, NLM, National Institutes of Health, Bethesda, Maryland, United States of America; Utrecht University, Netherlands

## Abstract

A fundamental observation of comparative genomics is that the distribution of evolution rates across the complete sets of orthologous genes in pairs of related genomes remains virtually unchanged throughout the evolution of life, from bacteria to mammals. The most straightforward explanation for the conservation of this distribution appears to be that the relative evolution rates of all genes remain nearly constant, or in other words, that evolutionary rates of different genes are strongly correlated within each evolving genome. This correlation could be explained by a model that we denoted Universal PaceMaker (UPM) of genome evolution. The UPM model posits that the rate of evolution changes synchronously across genome-wide sets of genes in all evolving lineages. Alternatively, however, the correlation between the evolutionary rates of genes could be a simple consequence of molecular clock (MC). We sought to differentiate between the MC and UPM models by fitting thousands of phylogenetic trees for bacterial and archaeal genes to supertrees that reflect the dominant trend of vertical descent in the evolution of archaea and bacteria and that were constrained according to the two models. The goodness of fit for the UPM model was better than the fit for the MC model, with overwhelming statistical significance, although similarly to the MC, the UPM is strongly overdispersed. Thus, the results of this analysis reveal a universal, genome-wide pacemaker of evolution that could have been in operation throughout the history of life.

## Author Summary

A central concept of evolution is Molecular Clock according to which each gene evolves at a characteristic, near constant rate. Numerous studies support the Molecular Clock hypothesis in principle but also show that the clock is indeed very approximate. Genome-wide comparative analysis of phylogenetic trees described here reveals a distinct, more general feature of genome evolution that we called Universal Pacemaker. Under this model, when the rate of evolution changes, the change occurs synchronously in many if not all genes in the evolving genome. In other words, the relative rates of gene evolution remain constant across long evolutionary spans: if a gene is slow relative to the rest of the genes in the given lineage, it is always slow, and if it evolves fast, it is always fast. We show here that the Universal Pacemaker model fits the available data much better than the traditional Molecular Clock model. These findings are compatible with the previously observed accelerations and decelerations of evolution in individual lineages but we show that synchronous, genome-wide change of evolutionary rates is a global feature of genome evolution that appears to pervade the entire history of life.

## Introduction

Genome-wide analysis of distances between orthologous genes in pairs of organisms from a broad range of taxa belonging to all three domains of life (bacteria, archaea and eukaryotes) revealed striking similarity between the distributions of these distances. All these distributions are approximately lognormal, span a range of three to four order of magnitude and are nearly identical in shape, up to a scaling factor [1]–[3]. Although many different explanations are possible of this remarkable conservation of evolutionary rate distribution across the entire spectrum of life, the simplest underlying model is that all genes evolve at approximately constant rates relative to each other, i.e. the changes in the gene-specific rates of evolution are strongly correlated genome-wide. This general model of evolution can be denoted Universal PaceMaker (UPM) of genome evolution: all genes in evolving genomes, in each evolving lineage, change their evolutionary rate (approximately) in unison although the pacemakers of different lineages need not to be synchronized.

The existence of UPM is compatible with the considerable amount of available data on fast-evolving and slow-evolving organismal lineages, primarily different groups of mammals [4], [5]. Conceivably, lineage-specific accelerations and decelerations of evolution can be caused by changes in the effective population size, and such rate changes are indeed expected to equally affect all genes in evolving genomes. The evolutionary rate has also been linked with other biological features of animals that are collectively denoted life history [5]. For instance, a genome-wide comparison of the evolutionary rates in the human and mouse lineages has shown that the number of fixed mutations per unit time is about twofold greater in rodents than it is in primates, with the implication that a lineage-specific, genome-wide change of evolutionary rate occurred after the separation of these lineages [6]. In the same vein, a genome-wide analysis of ratios between the evolutionary rates of orthologous genes in triplets of related bacterial, archaeal and mammalian species revealed near constancy of these ratios, with only a small percentage of gene-specific deviations that were attributed to functional diversification of individual genes [7]. A systematic study of densely populated phylogenetic trees for 44 mammalian genes has demonstrated clade-specific slowdown of evolution occurring independently in several orders including primates and whales [8]. Multiple studies of mitochondrial DNA evolution that used extensive samples from numerous taxa also detected consistent lineage-specific rates that differed by as much as an order of magnitude between animal taxa [9], [10]. However, in other analyses, striking differences between lineages in the relative rates of evolution of different genes have been discovered, casting doubt on the universality of lineage-specific rates, leading to the idea of ‘erratic evolution’ [11], [12].

The plausibility of the UPM notwithstanding, the genome-wide correlations between the evolutionary rates of individual genes also could be explained within the concept of molecular clock which is one of the central tenets of molecular evolution. In 1962 Zuckerkandl and Pauling discovered that the number of differences between homologous proteins is roughly proportional to the divergence time separating the corresponding species [13], [14]. This phenomenon became known as Molecular Clock (MC) and has been validated by multiple independent observations [15]–[18]. The MC is the basis of molecular dating whereby the age of an evolutionary event, usually the split between lineages (such as for example humans and chimpanzee), is estimated from the sequence divergence using calibration with dates known from fossil record [19]–[22]. From the phylogenetic point of view, when genes evolve along a rooted tree under the MC, branch lengths are proportional to the time between speciation (or duplication) events and the distances from each internal tree node to all descendant leaves are the same (ultrametric tree) up to the precision of the estimation (the latter being determined by sampling error which is inevitable in comparison of finite-length sequences).

Over the 50 years that elapsed since the seminal finding of Zuckerkandl and Pauling, the MC has been shown to be substantially overdispersed, i.e. the differences between the root to tip distances in many or most subtrees of a given tree usually greatly exceed the expectation from sampling error, under the assumption of a Poisson mutational process [23]–[26]. Notably, the overdispersion of the MC has been shown to be lineage-specific: the MC in lineages with large effective population sizes is overdispersed to a greater extent than the MC in lineages with small populations implying that deviations from the MC are controlled by selection [27]. The demonstration of the overdispersion of the MC inspired the relaxed MC model which is a compromise between an unconstrained tree with arbitrary branch lengths and an MC tree [28], [29]. Under the relaxed MC, the evolutionary rate is allowed to change from branch to branch but this change is presumed to be gradual so that related lineages evolve at similar rates. The relaxed MC model underlies most of the modern methods of molecular dating.

The strict MC implies that all orthologous genes present in a group of organisms and sharing the same evolutionary history evolve in a fully coherent manner even if at different rates. Indeed, if the divergence between gene sequences is solely determined by the divergence time and gene-specific evolution rate, phylogenetic trees reconstructed from different genes will have the same topology and nearly identical branch lengths up to a scaling factor which is equal to the relative evolution rate. Under the MC model, the differences between the corresponding branch lengths in different gene trees are due solely to the sampling error which arises from stochastic factors and is expected to be uncorrelated between trees. The relaxed MC model allows greater, non-random deviations in the lengths of corresponding branches but to our knowledge, the possibility that these evolution rate changes are correlated between genes has not been explicitly considered.

The MC implies the constancy of gene-specific relative evolution rates, with deviations caused by overdispersion. However, the inverse is not true: the deviations of the absolute evolution rates from the clock could be arbitrarily high (hence no MC) but, if they apply to all genes in the genome to the same degree, the relative evolutionary rates would remain approximately the same throughout the entire course of evolution and in all lineages. In other words, the conservation of the evolutionary rate distribution follows from a model of evolution that is more general and less constrained than the MC, namely the UPM model.

Here we sought to determine which of the two models of gene evolution, the MC and or the UPM, better fits the empirical data. To this end, we performed comparative analysis of phylogenetic trees for a genome-wide set of prokaryotic gene families and compared the goodness of fit for the two models. The results show that the UPM model is a better fit than the MC model for the evolution of prokaryotes. These findings are compatible with the previously observed accelerations and decelerations of evolution in individual evolving lineages. However, we show that synchronous, genome-wide change of evolutionary rates is a universal trend of genome evolution that appears to pervade the entire history of life.

## Results/Discussion

### Fitting individual gene trees to the supertree

**Figure 1:**
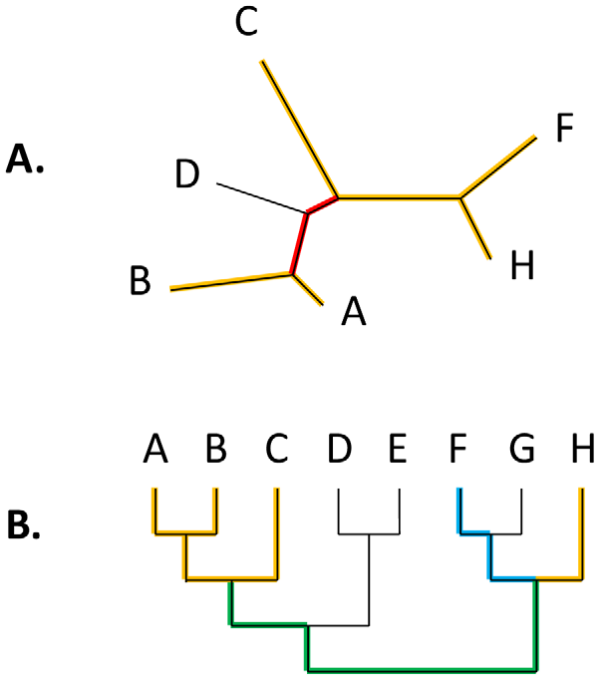
Gene trees and the supertree. A. A gene tree (GT). After the comparison with the supertree (ST), the GT is reduced to the maximum agreement subtree (MAST, highlighted in yellow). The reduced GT edge highlighted in red corresponds to two edges in the original GT. B. Supertree (ST). Mapping of the reduced GT onto the ST is highlighted; two sections of ST that consist of multiple edges mapping to a single edge of the reduced GT are highlighted in blue and green, respectively.

Our data set consisted of the “forest” of phylogenetic trees reconstructed for 6901 orthologous gene families representing 41 archaeal and 59 bacterial genomes [30] (see Supporting Text S1). Although horizontal gene transfer is widespread in the evolution of prokaryotes [31], [32], the tree-like statistical trend is detectable in the genome-wide data set and moreover dominates the evolution of (nearly) ubiquitous gene families [30], [33]. We encapsulate this trend in a rooted supertree (ST) that reflects the prevalent vertical descent in the evolution of archaea and bacteria (see Supporting Text S1). Each individual original gene tree (GT) is compared to the ST and reduced to the maximum agreement subtree (MAST), i.e. the largest set of leaves whose phylogeny fits the ST topology. Removal of discordant nodes and edges leads to collapse of several edges of the original GT into a single edge (Figure 1); then, the length of the newly created GT edge is the sum of the original contributing GT edges. Likewise, when a GT is mapped to the ST, several adjacent ST edges could correspond to a single edge in the reduced GT, forming a composite edge.

Under both the MC and the UPM models, we assume that the lengths of the ST edges determine the expected lengths of the corresponding GT edges. For the MC model, edge lengths correspond to time intervals between speciation events, the ST is strictly ultrametric, and gene-specific evolutionary rates are measured in substitutions per site per time unit. Under the UPM model, edge lengths represent arbitrarily defined “ticks” of the universal pacemaker (internal time), and gene-specific evolutionary rates are measured in substitutions per site per pacemaker unit of internal time. Formally:
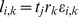
where *l_i,k_* is the length of the *i*-th edge of the *k*-th GT, *t_j_* is length of the *j*-th (possibly composite) ST edge corresponding to the *i*-th edge of the *k*-th GT, *r_k_* is the gene-specific evolution rate, and ε*_i,k_* is the multiplicative error factor for the given edge. We further assume that the error is random, independent for branches both within and between GTs, and comes from a lognormal distribution with the mean of 1 and an arbitrary variance, translating to a model with an additive normally distributed deviation in the logarithmic scale. Because the distributions of evolutionary rates tend to follow symmetric bell-shaped curves in log scale [3], [34], the assumption of a multiplicative, log-normally distributed deviation seems natural.

First, we seek to find the set of ST edge lengths ***t*** and gene rates ***r*** that provides the best fit to the entire set of GTs. Under the assumption of a normally distributed deviation, the likelihood function for the set of GTs given ***t*** and ***r*** is
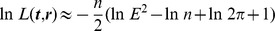
where *n* is the total number of edges in the set of GTs and *E* ^2^ is the sum of squares of deviations between the expected and observed edge lengths in the logarithmic scale:
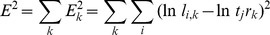
where the summation for *i* is done over the edges of a given GT and the summation for *k* is done over all GTs (see Supporting Text S2). Thus, finding the maximum likelihood solution for {***t***, ***r***} is equivalent to finding the minimum of *E* ^2^. For the MC model, the ST edge lengths ***t*** are constrained by the ultrametricity requirement, whereas for the UPM model, ST edge lengths are unconstrained.

For the analyzed set of 100 genomes, there is a choice of several possible ST topologies, produced using different methods (see Methods and Supporting Figure S1). We mapped all original GTs onto each of these STs and obtained reduced GTs that corresponded to the respective MASTs. The GTs that yielded MASTs with fewer than 10 leaves were discarded. The ST topology derived from the concatenated alignments of ribosomal proteins provided the maximum total number of leaves in the resulting set of reduced GTs and accordingly was chosen for further analysis. Altogether, we obtained 2294 reduced GTs with MAST size greater or equal to 10 species including 44,889 leaves and 82,896 edges. This set of trees was fit to an ultrametricity-constrained ST (MC model) and an unconstrained ST (UPM model) (Table 1, see Supporting Text S3 for details).

**Table 1:** Comparison of the Molecular Clock and Universal Pacemaker models of genome evolution.

	MAST≥30		MAST≥20		MAST≥10	
	MC	UPM	MC	UPM	MC	UPM
**Number of trees**	246		967		2,294	
**Number of leaves**	9,134		26,441		44,889	
**Number of edges**	17,530		49,981		82,896	
***E^2^***	10,656.3	10,197.8	36,139.7	35,065.0	68,260.8	66,626
**r.m.s.d., ln units**	0.7797	0.7627	0.8503	0.8376	0.9074	0.8965
**r.m.s.d., factor**	2.1808	2.1441	2.3404	2.3108	2.4780	2.4510
***ΔAIC***	573.0	0	1,310.8	0	1810.8	0
**Relative likelihood weight**	10^−125^	1	10^−285^	1	10^−393^	1
***ΔBIC***	−196.4	0	437.7	0	887.6	0

### The goodness of fit between gene trees and the supertree under the molecular clock and universal pacemaker models of evolution

We then compared the MC and UPM models in terms of the goodness of fit to the data. Obviously, the residual sum of squares is lower for the UPM model because it involves independent optimization of all 198 ST edge lengths, whereas under the MC model the edge lengths are subject to 99 ultrametricity constraints. To account for the difference in the numbers of degrees of freedom, we employed the Akaike Information Criterion (AIC) and the Bayesian Information Criterion (BIC) to compare the MC and UPM models. Under the assumption of normally distributed deviations:
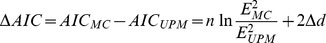
and
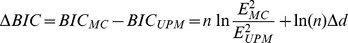
where *E* ^2^ *_MC_* and *E* ^2^ *_UPM_* are the residual sums of squares for the MC and UPM models, respectively, *n* is the total number of GT edges and Δ*d* is the difference in the number of parameters optimized in the process of fitting (in our case Δ*d* = −99). Because lower AIC values correspond to better quality of fit, negative Δ*AIC* would indicate preference for the MC model whereas a positive Δ*AIC* would indicate support for the UPM model. The relative likelihood weight of the suboptimal model can be estimated as 1/exp(|Δ*AIC*|/2). The same calculations were repeated for smaller, more conservative subsets of gene families with MAST>20 and MAST>30 and also using BIC to compare the fit to the UPM and MC models (Table 1).

**Figure 2:**
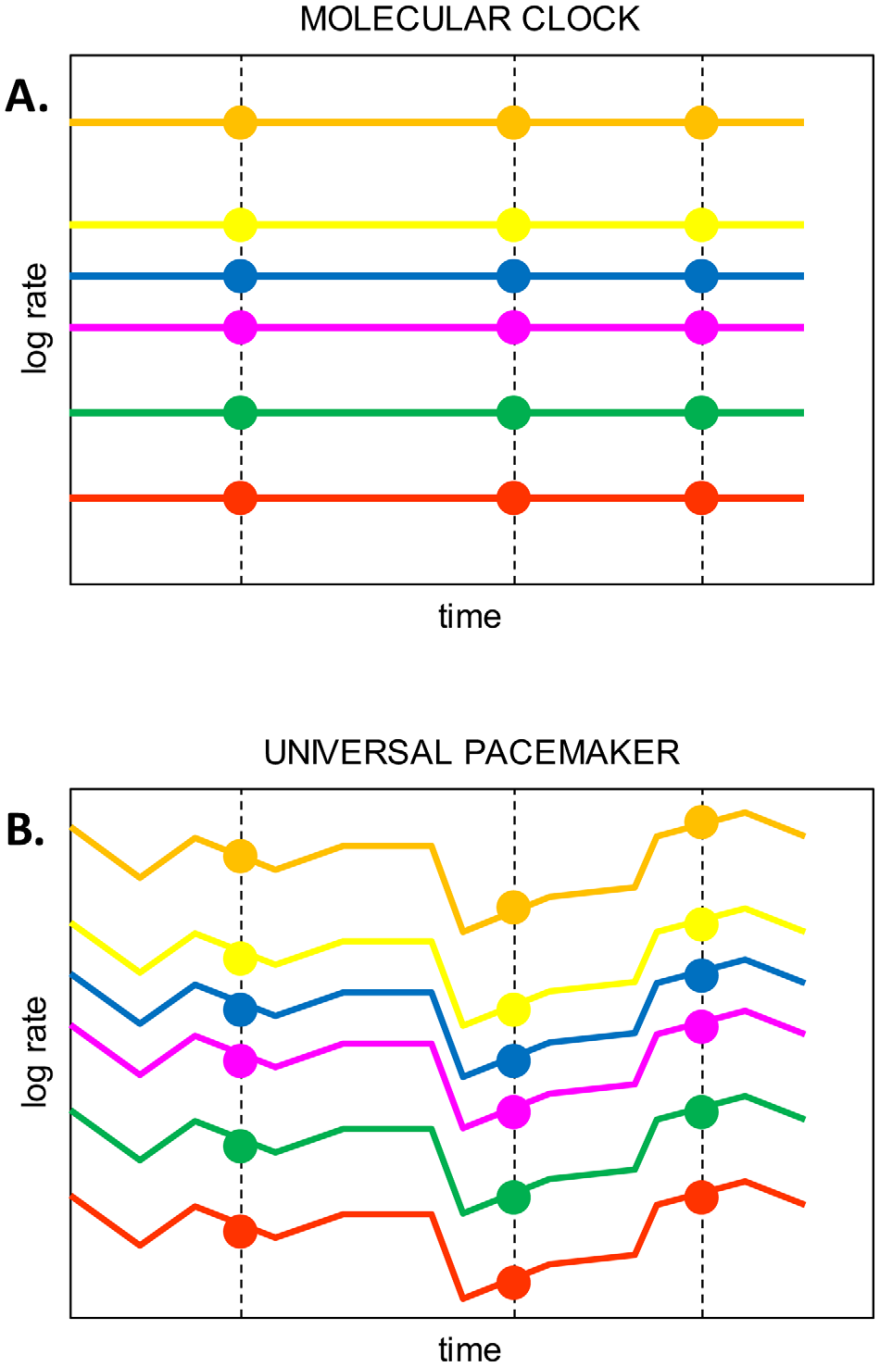
The Universal Molecular Clock and Universal Pacemaker models of genome evolution. A. Under the Molecular Clock model, gene-specific evolution rates (colored lines) remain constant; at any point in time (shown as dots), the relative rates of gene evolution are also constant. B. Under the Universal Pacemaker model, gene-specific evolution rates can change arbitrarily but by the same amount across the entire genome; at any point in time, the relative rates of gene evolution remain constant.

Overall, the results presented in Table 1 reveal overwhelming support of the UPM model over the MC model. The only exception is the *ΔBIC* value for MAST>30 that weakly supports the MC model. This outcome is predictable given the much larger number of parameters in the UPM model, the small number of trees in this subset and the heavier penalty that BIC imposes on parameter-rich models [35]. Thus, the results show that the evolutionary rates tend to change synchronously for the majority (if not all) of the genes in evolving genomes although the rate of the UPM relative to the astronomical time differs for different lineages. The results of this analysis show that the apparent genome-wide constancy of the relative rates of gene evolution across vast spans of life's history (Figure 2A) is not a trivial consequence of MC but at least in part results from a distinct, fundamental evolutionary phenomenon, the UPM (Figure 2B).

**Figure 3:**
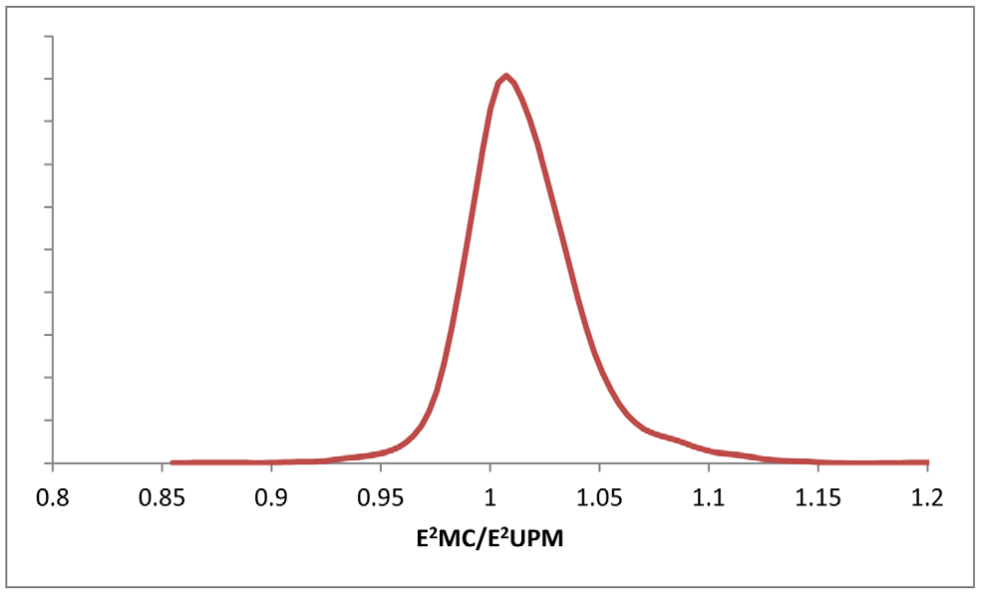
The distribution of the *E* ^2^ *_MC,k_*/*E* ^2^ *_UPM,k_* ratios for 2294 gene families. The curve was smoothed using the Gaussian-kernel method.

The difference between the UPM and MC models is highly significant but small in magnitude. Root mean square deviation (r.m.s.d.) of GT edges from the expectations derived from UMP ST is large (a factor of 2.45) and only slightly less that the r.m.s.d for the MC ST (a factor of 2.48). Thus, similar to MC, the UPM appears to be substantially overdispersed. To assess the robustness of the finding that UPM fits the GTs better than MC, we isolated the contributions of individual trees to the *E* ^2^ *_MC_* and *E* ^2^ *_UPM_* (*E* ^2^ *_MC,k_* and *E* ^2^ *_UPM,k_* respectively), took 1000 bootstrap samples of the set of GTs and computed Δ*AIC* values for each sample. All 1000 Δ*AIC* values obtained for the resampled sets were positive (in the range of 1511 to 2147), providing 100% support to the superiority of the UPM model and ensuring that this result is consistent for the majority of the GTs and is not determined by a small number of strongly biased trees (see Supporting Text S3 and Supporting Figure S2 for details). The distribution of the *E* ^2^ *_MC,k_*/*E* ^2^ *_UPM,k_* ratios (Figure 3) shows a strong bias toward values greater than unity (73% of the GTs), supporting the robustness of this result.

**Figure 4:**
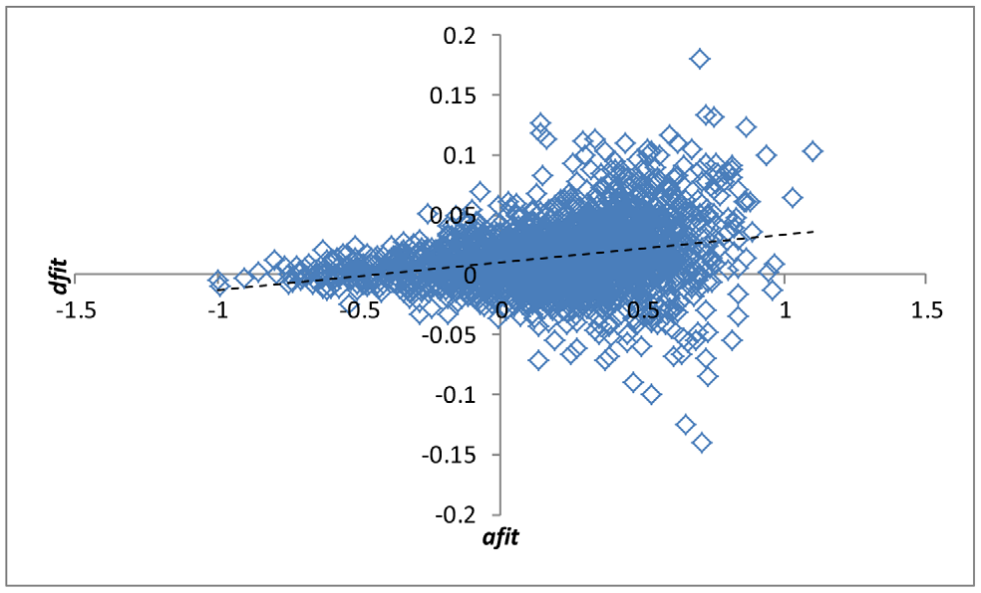
Relative goodness of fit for the UPM vs the MC model (*dfit*) plotted against the average goodness of fit (*afit*). *dfit*: log_10_(*E* ^2^ *_MC,k_*/*E* ^2^ *_UPM,k_*). *afit*: −(log_10_(*E* ^2^ *_MC,k_*)+log_10_(*E* ^2^ *_UPM,k_*))/2.

The *E* ^2^ *_MC,k_*/*E* ^2^ *_UPM,k_* ratio characterizes the degree to which the *k*-th GT favors the UPM model. Linear model analysis shows that this value is significantly and independently influenced by the average goodness of fit to the ST (p-value ≪0.001; Figure 4), the fraction of the original GT leaves remaining in the MAST with ST (p-value ≪0.001; Supporting Figure S3) and the number of the original GT leaves (p-value ≪0.001; Supporting Figure S3). Thus, the GTs that retain a greater number of leaves in the MAST, fit the ST better and are wider distributed among prokaryotes, typically show the strongest preference for the UPM model over the MC model. These three factors together explain ∼9% of the variance in ln(*E* ^2^ *_MC,k_*/*E* ^2^ *_UPM,k_*). Neither the relative evolution rate nor the functional class of the gene significantly impact the degree of preference of UPM over MC (see Supporting Text S3 and Supporting Figure S3 for details). Interpreting these findings in terms closer to biology, widely-distributed genes that are subject to relatively little horizontal transfer or sporadic changes of evolution rate that reduce the fit to ST appear to make the greatest contribution to the UPM. These observations imply that the UPM is indeed a fundamental feature of genome evolution, at least in prokaryotes.

**Figure 5:**
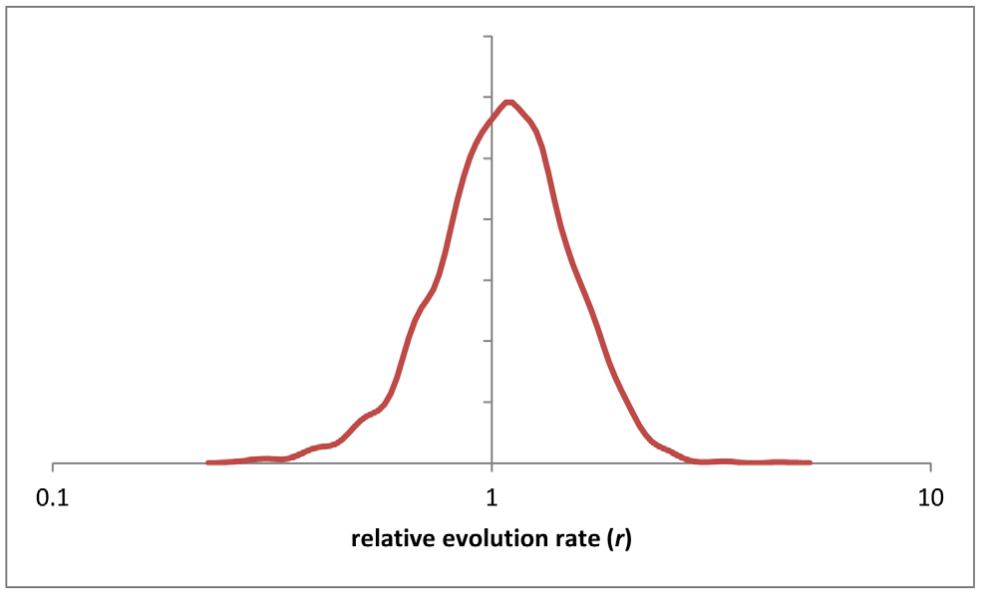
The distribution of the relative evolution rates (*r_k_* values) for 2294 gene families obtained by fitting gene trees to the UPM (unconstrained) supertree. The curve was smoothed using the Gaussian-kernel method.

The distribution of estimated relative evolution rates (Figure 5) spans values within a range slightly greater than an order of magnitude (0.26 to 4.58). This range is considerably more narrow than the range of rates measured over short evolutionary distances [3], [34]. Accelerations and decelerations of the UPM are likely to average out over long intervals of evolution, reducing the observed differences between genes.

### How many pacemakers are possible?

A logical extension of the UPM is a Multiple PaceMakers (MPM) whereby a number of uncorrelated pacemakers ‘guide’ their own sets of trees. In the extreme case, the number of PMs is equal to the number of GTs so that the individual GTs would be completely uncorrelated. We sought to explore this case in order to determine how well such a degenerate MPM (dMPM) model fits the data compared to the UPM and MC.

Formally, under the basic assumptions of this work, the log likelihood of dMPM is infinite because the *E* ^2^ value is estimated as the sum of squared differences between the observed and the expected edge lengths. Under dMPM, each edge is equal to its own expectation sothat *E* ^2^ = 0. However, this logic assumes that the tree edge length is measured precisely and is not subject to any error, whereas the *E* ^2^ value is dominated by deviations of individual GTs from the universal standard (MC or UPM). This assumption is obviously unrealistic, so to assess the likelihood of the dMPM, one needs to introduce the edge length estimate error explicitly.

To obtain the lower limit on the *E* ^2^ value induced by the inherent sampling fluctuations, one should note that the sum of the lengths of the 49,981 edges in 967 trees (MAST size ≥20) is 13,018.5 (substitutions per site), on average 0.26 per edge. With the typical prokaryotic protein length being ∼200 amino acids [36], this translates into the average of ∼52 substitutions per tree branch. Assuming that substitutions are generated by a Poisson-type random process, one expects the standard deviation of approximately 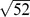 and the “mean” error of the observed value on the order of (52+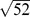)/52 = 1.14 or 0.13 log units per branch. Multiplying the square of this value by 49,981 edges, we obtain the *E* ^2^ value estimate of 843.0, much lower than 35065.0 for UPM. It should be noted that the use of the average gene length and the average number of substitutions per branch comprises the ‘best-case scenario’ because variations in both would necessarily introduce larger deviations which would increase the *E* ^2^ value.

To calculate the Δ*AIC* value, one needs to obtain the difference in the degrees of freedom between the UPM and dMPM models. The UPM model uses the estimates of 198 individual edge lengths in one UPM tree plus 967 GT rates; the dMPM model requires 967±198 edge length estimates and no GT rates, yielding Δ*d* = −190,301.

Plugging these values into the equation for Δ*AIC*, one gets the difference of −194,269 in the UPM-dMPM comparison. Thus, the dMPM model is less likely than the UPM model by 83,370 orders of magnitude, an obvious indication that the assumption of completely uncorrelated rate changes does not fit the data. More specifically, the data would support no more than 476 pacemakers for 967 GTs under ideal conditions (each GT follows its PM perfectly, so the *E* ^2^ value remains to be solely determined by sampling fluctuations). Thus, the actual number of distinct pacemakers is expected to be much lower.

### Concluding remarks

The results of the genome-wide comparison of phylogenetic trees of prokaryote genes described here show that the UPM model fits the data substantially better than the MC model. These findings have no bearing on the validity of the MC but show that a more general conservation principle (the UPM) is sufficient to explain the observed correlations between gene-specific evolutionary rates. It seems a natural possibility that UPM is instigated by shifts in population dynamics of evolving lineages, with changes affecting all genes in the same direction and to a similar degree. In principle, UPM reflects the well-known phenomenon of lineage-specific acceleration-deceleration of evolution. However, to our knowledge, the previous studies on this phenomenon have focused primarily on mammals and to a lesser extent other vertebrates [4], [5]. Here we show that the UPM can explain the correlations between the evolutionary rates of prokaryote genes on the whole genome scale and over time intervals that span effectively the entire history of life on earth. The discovery of the UPM opens up several areas of further inquiry. We show here that an unconstrained model of evolution (dMPM) does not fit the data but it remains to be determined whether or not distinct pacemakers govern the evolution of different classes of genes. The biological connotations of the UPM are of major interest. Mapping UPM shifts to specific stages of the evolution of life, changes in the life style and population structure of organisms as well as to the geological record could become an important direction of future research.

## Methods

### Supertrees and Maximum Agreement Subtrees

Three distinct supertrees (STs) were tested for the purpose of representing the vertical inheritance trend in the analyzed set of GTs. The first supertree (ST_1_) was from [30] (originally computed using the CLANN program [37]; the second supertree (ST_2_) was computed using the quartet supertree method [38] for all species quartets in the complete set of GTs the third supertree (ST_3_) was derived from a tree of concatenated sequences of (nearly) universal ribosomal proteins [39]. Maximum Agreement Subtrees (MAST) between the supertree (ST) and any given gene tree (GT) were computed using the *agree* program of the PAUP* package [40]. The set of MASTs with the analyzed GTs was computed for each of these STs, yielding a total of 43,068 MAST leaves for ST_1_, 43,411 MAST leaves for ST_2_ and 44,889 MAST leaves for ST_3_ (MAST ≥10 for each ST). Accordingly, ST_3_ was used for all further analyses as the topology that best represented the entire set of GTs.

To perform the LS optimization of the ST edge lengths and the GT relative evolution rates, we used the function *fmin_slsqp()* that is part of the *scipy.optimize* package of Python which minimizes a function using sequential least squares programming. The function also adopts a set of constraints that are necessary for the calculation. In both the MC and the UPM models, both the ST edges and the GT rates were constrained to positive values. For the UPM model, the distances from a node to any leaf in a subtree under that node were set equal for all subtrees. It can be shown by induction that this constraint implies an ultrametric tree. Thus, we have a constraint for every internal node; in a rooted binary tree with *m* leaves, there are *m*−1 such nodes.

### Maximum likelihood estimate for the supertree edge lengths and gene evolution rates

Consider a rooted supertree (ST) with a fixed topology. The ST encompasses a set of edges ***e*** defined by the ST topology and a set of unknown edge lengths ***t***. Consider a set of unrooted GTs reduced to MAST with the given ST. Each GT encompasses a set of edges with known edge lengths and an unknown gene-specific evolution rate (***b*** *_k_*, ***l*** *_k_* and *r_k_* for the *k*-th GT, respectively). Each edge of each GT uniquely maps to an ST path ***e*** *_j_*, that is a subset of adjacent edges in the ST (*b_k,i_*≡***e*** *_j_* where ***e*** *_j_*⊆***e*** for the *i*-th edge of the *k*-th GT).

Let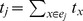 be the length of the path ***e*** *_j_*. We assume that the length of the *i*-th edge of the *k*-th GT is related to the length of the corresponding ST path ***e*** *_j_*:
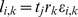
where ε*_i,k_* is the multiplicative deviation factor for the given edge. We further assume that the deviation is random, independent for branches both within and between GTs, and comes from a lognormal distribution with the mean of 1 and an arbitrary variance, translating to a model with an additive normally distributed deviation in the logarithmic scale (i.e. ln ε*_i,k_*∼*N*(0,σ^2^)).

Given ***t*** and ***r***, the expectation for the logarithm of the length of the *i*-th edge of the *k*-th GT is:
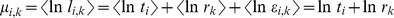
and the likelihood of observing the length *l_i,k_* is:
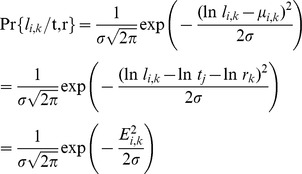
where *E* ^2^ *_i,k_* = (ln *l_i,k_*−ln *t_j_*−ln *r_k_*)^2^. For all observed edge lengths in all GTs (***l***), the likelihood function is
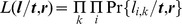
In the logarithmic scale:
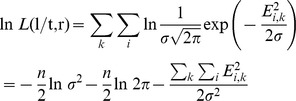
where *n* is the total number of GT edges (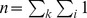). Designating the residual sum of squares 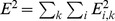 and substituting the estimate for σ^2^

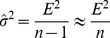
for large *n*, we obtain:
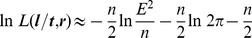
Because *n* is constant for a given data set, finding the maximum of *L*(***l*** | ***t***,***r***) is equivalent to finding the minimum of *E* ^2^.

### Least squares optimization procedure

Least Squares (LS) is called linear if the residuals are linear for all unknowns. Linear LS can be represented in a matrix format which has a closed form solution (given that the columns of the matrix are linearly independent). However, our formulation requires taking logs over sums of unknowns in the case where a GT edge corresponds to a path in ST (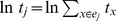). Then, the problem becomes non-linear with respect to LS and can be solved only using numerical algorithms where the solution is obtained by iteratively refining the parameter values. This approach requires supplying initial values for the parameters. The goodness of the initial value estimation is critical for the convergence time of the iterative method and the risk of being trapped in local maximum points. We employed the following strategy for determining the initial values: For each ST edge, we computed the mean value of the sum over all GT edges that uniquely correspond to the given edge. Therefore, if we assign one gene a specific rate value (e.g. the length of some edge), we obtain initial rate values for all genes. It can be easily shown that, if there are no errors in rates (i.e. σ^2^ = 0), the above procedure yields the accurate (ML) values for all unknowns.

## Supporting Information

Figure S1 MC and UPM optimization of the supertree branch lengths.
(PDF)

Figure S2 Distribution of the Δ*AIC* values for 1000 bootstrap samples (the curve was obtained by Gaussian-kernel smoothing of the individual data points). The red line indicates the Δ*AIC* value for the original set of GTs (1310.8).
(PDF)

Figure S3 A: Relative goodness of fit for the UPM vs the MC model (*dfit*) plotted against the fraction of original GT leaves retained in MAST (*mg*). B: Relative goodness of fit for the UPM vs the MC model (dfit) plotted against the average goodness of fit (afit). C: Relative goodness of fit for the UPM vs the MC model (dfit) plotted against the relative evolution rate (r).C.
(PDF)

Text S1 Supertree (ST_3_) topology (Newick format).
(DOCX)

Text S2 Maximum Likelihood estimates for the supertree edge lengths and gene evolution rates.
(DOCX)

Text S3 Goodness of fit for the MC and UPM models: bootstrap analysis and dependence on evolutionary and functional charateristics of gene families.
(DOCX)
