# Supplementary material for: Universal Pacemaker of Genome Evolution
Source: PLoS Comput Biol. 2012 Nov 29;8(11):e1002785. doi: 10.1371/journal.pcbi.1002785 (PMC3510094; doi:10.1371/journal.pcbi.1002785)
Supplement: Figure S1 — MC and UPM optimization of the supertree branch lengths. (PDF) [file pcbi.1002785.s001.pdf]

MC-constrained (ultrametric) supertree

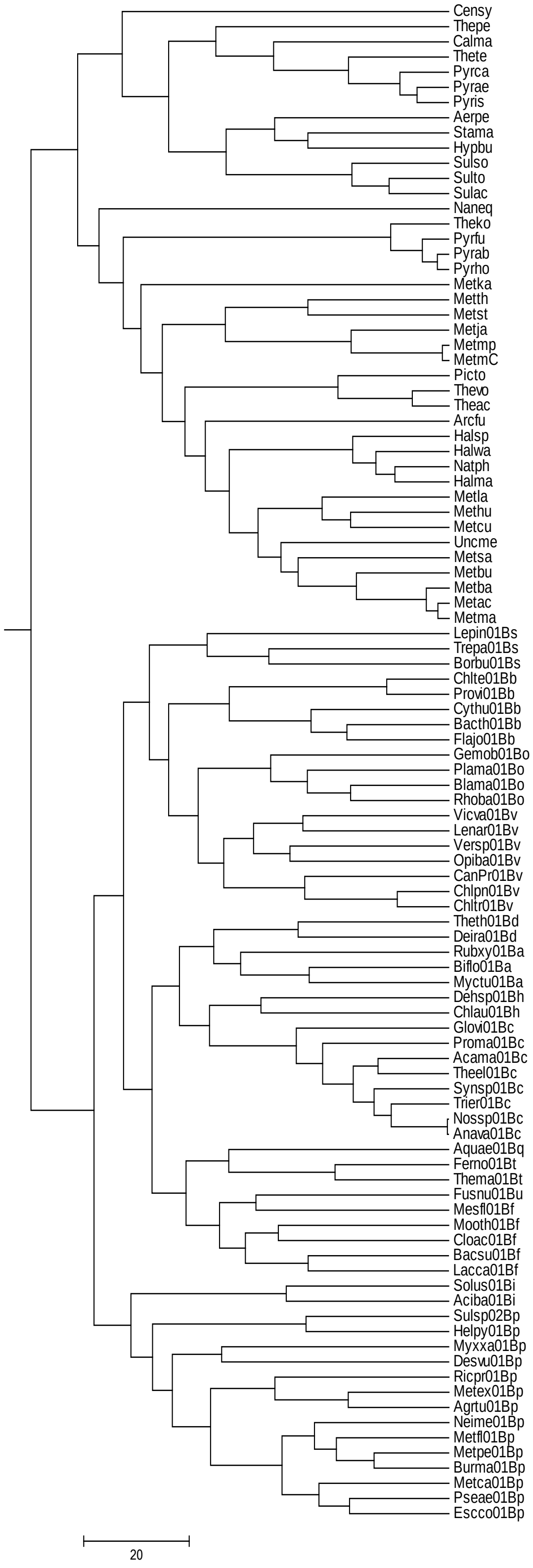

Unconstrained (UPM) supertree

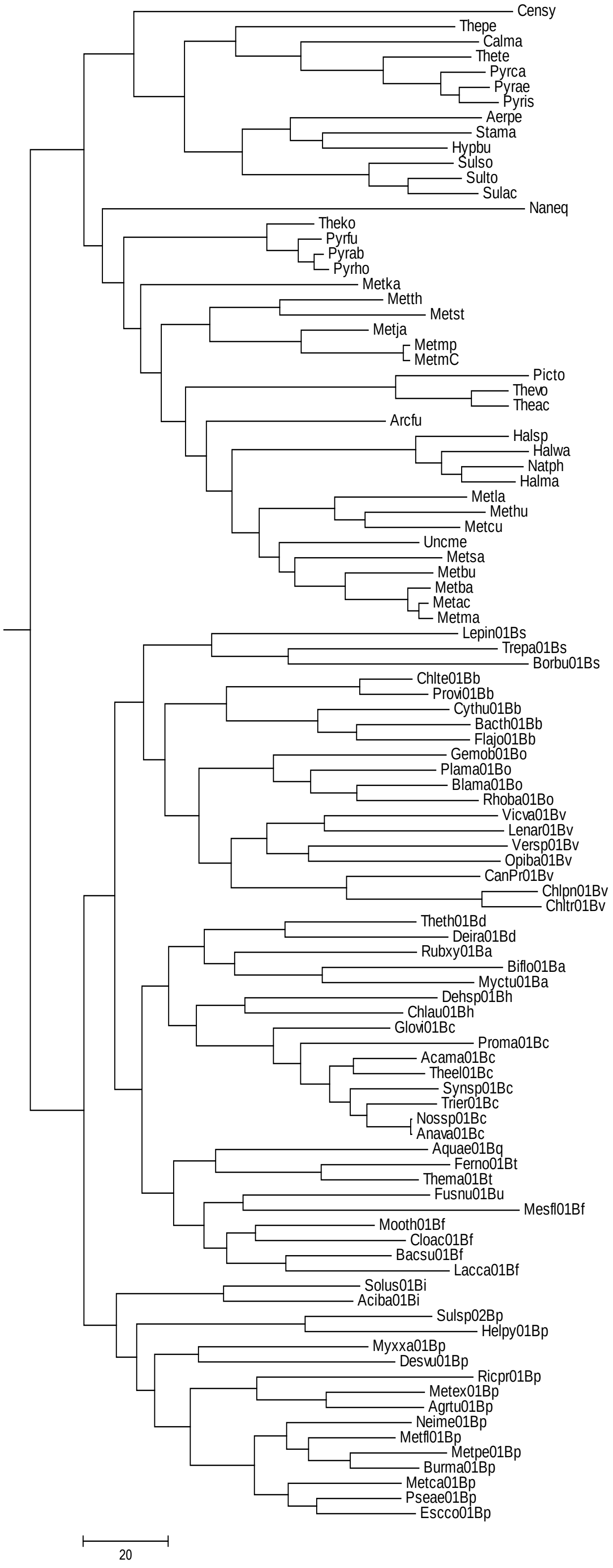

MC-optimized supertree, Newick format.

(((Censy:61.8869,((Thepe:44.1285,(Calma:33.1595,(Thete:18.9522,(Pyrca:9.1778,(Pyrae:5.9390,Pyris:5.
9390):3.2388):9.7744):14.2072):10.9690):8.9634,((Aerpe:33.0161,(Stama:26.7236,Hypbu:26.7236):6.292
5):9.1865,(Sulso:18.3712,(Sulto:11.3010,Sulac:11.3010):7.0703):23.8314):10.8892):8.7951):8.4681,(Nan
eq:66.3254,((Theko:11.0283,(Pyrfu:5.0095,(Pyrab:2.1686,Pyrho:2.1686):2.8409):6.0188):50.7054,(Metka
:58.3823,(((Metth:26.7240,Metst:26.7240):15.6488,(Metja:18.5184,(Metmp:1.2279,MetmC:1.2279):17.29
05):23.8544):11.9734,((Picto:21.0763,(Thevo:6.9803,Theac:6.9803):14.0960):28.9880,(Arcfu:46.2026,((H
alsp:18.2850,(Halwa:13.8666,(Natph:10.2491,Halma:10.2491):3.6175):4.4184):23.3907,((Metla:24.0772,(
Methu:18.6710,Metcu:18.6710):5.4062):12.1627,(Uncme:31.9038,(Metsa:28.6607,(Metbu:17.6049,(Metb
a:4.3497,(Metac:2.1720,Metma:2.1720):2.1777):13.2552):11.0558):3.2431):4.3362):5.4357):4.5269):3.86
17):4.2820):4.0360):3.3514):4.5918):4.0296):8.8492,((((Lepin01Bs:45.8589,(Trepa01Bs:34.1513,Borbu0
1Bs:34.1513):11.7076):10.9256,(((Chlte01Bb:11.7560,Provi01Bb:11.7560):29.8532,(Cythu01Bb:26.1225,
(Bacth01Bb:19.3430,Flajo01Bb:19.3430):6.7795):15.4867):11.5212,((Gemob01Bo:33.7971,(Plama01Bo:
26.8322,(Blama01Bo:18.6302,Rhoba01Bo:18.6302):8.2020):6.9649):13.7445,(((Vicva01Bv:27.6823,Lena
r01Bv:27.6823):9.3475,(Versp01Bv:30.1312,Opiba01Bv:30.1312):6.8986):5.6725,(CanPr01Bv:27.3706,(
Chlpn01Bv:9.8091,Chltr01Bv:9.8091):17.5615):15.3316):4.8393):5.5889):3.6540):4.8775,((((Theth01Bd:2
8.5267,Deira01Bd:28.5267):16.0194,(Rubxy01Ba:39.4497,(Biflo01Ba:26.4593,Myctu01Ba:26.4593):12.9
904):5.0964):6.5052,((Dehsp01Bh:35.5921,Chlau01Bh:35.5921):9.7385,(Glovi01Bc:28.8885,(Proma01Bc
:23.8659,((Acama01Bc:13.3682,Theel01Bc:13.3682):4.7123,(Synsp01Bc:14.1482,(Trier01Bc:10.8841,(N
ossp01Bc:0.2118,Anava01Bc:0.2118):10.6723):3.2641):3.9323):5.7853):5.0227):16.4421):5.7207):5.194
8,((Aquae01Bq:41.6984,(Ferno01Bt:21.5559,Thema01Bt:21.5559):20.1425):8.0988,((Fusnu01Bu:36.535
3,Mesfl01Bf:36.5353):6.8947,((Mooth01Bf:32.2905,Cloac01Bf:32.2905):6.2299,(Bacsu01Bf:26.5997,Lacc
a01Bf:26.5997):11.9207):4.9097):6.3671):6.4489):5.4159):5.6010,((Solus01Bi:30.8122,Aciba01Bi:30.812
2):29.4297,((Sulsp02Bp:27.0372,Helpy01Bp:27.0372):29.0993,((Myxxa01Bp:43.0730,Desvu01Bp:43.073
0):9.3309,((Ricpr01Bp:32.9890,(Metex01Bp:19.0928,Agrtu01Bp:19.0928):13.8962):12.1896,((Neime01B
p:25.4812,(Metfl01Bp:21.3187,(Metpe01Bp:14.1583,Burma01Bp:14.1583):7.1604):4.1624):6.1640,(Metc
a01Bp:24.6509,(Pseae01Bp:18.8580,Escco01Bp:18.8580):5.7928):6.9943):13.5334):7.2253):3.7327):4.1
054):7.0210):11.9412);

UPM-optimized supertree, Newick format

(((Censy:89.0259,((Thepe:51.4890,(Calma:41.7386,(Thete:20.5655,(Pyrca:10.4465,(Pyrae:7.0525,Pyris:
9.1721):4.3686):13.5020):19.3299):15.3357):12.0650,((Aerpe:44.9282,(Stama:34.9028,Hypbu:29.2652):7
.5422):11.3161,(Sulso:19.7941,(Sulto:12.5176,Sulac:16.4295):9.1575):29.7845):13.5787):11.9140):11.69
70,(Naneq:99.2062,((Theko:11.0680,(Pyrfu:5.4038,(Pyrab:2.1754,Pyrho:3.4121):3.7090):7.3922):33.640
2,(Metka:50.9979,(((Metth:24.1644,Metst:34.2067):16.4281,(Metja:15.7372,(Metmp:1.5000,MetmC:1.467
7):23.9853):21.4609):11.3870,((Picto:31.1798,(Thevo:8.6051,Theac:8.6739):17.7942):49.3736,(Arcfu:42.
0494,((Halsp:21.7838,(Halwa:20.4254,(Natph:14.4531,Halma:12.6005):4.7011):6.1063):43.1505,((Metla:
31.0090,(Methu:28.2125,Metcu:22.2977):7.1172):17.7435,(Uncme:32.8120,(Metsa:34.5754,(Metbu:20.61
89,(Metba:5.2481,(Metac:2.2004,Metma:3.2066):2.6050):14.7119):11.8231):3.6558):4.7029):6.4194):5.9
909):4.8941):5.7240):4.8166):3.9425):5.0274):4.3704):12.6121,((((Lepin01Bs:57.8117,(Trepa01Bs:49.14
54,Borbu01Bs:56.3925):17.9829):16.0285,(((Chlte01Bb:12.2697,Provi01Bb:16.0112):31.3288,(Cythu01B
b:30.8345,(Bacth01Bb:26.6925,Flajo01Bb:26.5281):9.1256):21.4746):14.4231,((Gemob01Bo:40.5998,(Pl
ama01Bo:29.4117,(Blama01Bo:21.2192,Rhoba01Bo:28.5604):10.8598):8.7642):17.4646,(((Vicva01Bv:4
0.7170,Lenar01Bv:42.1258):13.5515,(Versp01Bv:46.7282,Opiba01Bv:45.0404):9.7755):8.3447,(CanPr01
Bv:31.3328,(Chlpn01Bv:13.0385,Chltr01Bv:13.8949):31.8389):27.0921):7.5969):8.0290):5.0101):6.7397,(
(((Theth01Bd:30.7799,Deira01Bd:38.2666):18.9921,(Rubxy01Ba:42.7450,(Biflo01Ba:42.3532,Myctu01Ba
:35.5812):20.5856):7.1378):8.3914,((Dehsp01Bh:45.1610,Chlau01Bh:37.0665):11.3927,(Glovi01Bc:27.3
297,(Proma01Bc:40.5533,((Acama01Bc:14.7899,Theel01Bc:16.7404):5.5897,(Synsp01Bc:20.6511,(Trier
01Bc:16.3717,(Nossp01Bc:0.2758,Anava01Bc:0.2406):10.3218):3.8772):4.8466):6.8180):6.4049):18.113
9):6.5438):6.2510,((Aquae01Bq:49.7884,(Ferno01Bt:30.2272,Thema01Bt:22.8174):24.8129):9.8613,((Fu
snu01Bu:43.8391,Mesfl01Bf:64.8579):9.1732,((Mooth01Bf:27.9007,Cloac01Bf:35.2075):6.7564,(Bacsu01
Bf:24.7567,Lacca01Bf:38.3900):13.8760):5.3383):7.1576):7.4726):6.3673):7.2299,((Solus01Bi:32.0055,A

ciba01Bi:30.4161):25.2051,((Sulsp02Bp:29.7525,Helpy01Bp:40.3668):39.5585,((Myxxa01Bp:39.8124,Desvu01Bp:46.1556):10.2976,((Ricpr01Bp:50.8076,(Metex01Bp:23.0949,Agrtu01Bp:22.8619):16.3410):15.6247,((Neime01Bp:29.2603,(Metfl01Bp:20.3604,(Metpe01Bp:22.6169,Burma01Bp:16.1408):9.8286):5.1449):7.5865,(Metca01Bp:26.5222,(Pseae01Bp:19.7872,Escco01Bp:23.2288):6.6808):7.9313):15.0439):8.3809):4.2225):4.7923):7.6533):12.6121);

GT relative evolution rates optimized under the UPMmodel.

GT	r	GT	R	GT	r	GT	r
COG0201	0.76659	COG0043	1.02903	COG2007	0.98454	COG1608	1.30463
COG0086	0.74428	COG0214	0.40733	COG1853	1.39015	COG2003	1.29369
COG0085	0.57485	COG0537	0.65288	COG1471	0.94314	COG0710	1.29338
COG0480	0.62635	COG1690	0.73195	COG0290	0.77355	COG1048	0.73188
COG0504	0.78924	COG0382	1.07937	COG1594	0.34193	COG1042	1.15700
COG0093	0.41826	COG1269	1.47665	COG0058	1.10634	COG1625	1.29514
COG0468	0.55648	COG0089	0.89662	COG0190	0.86204	COG0260	1.23970
COG0525	0.83753	COG0473	0.85938	COG0852	1.08477	COG0199	0.77174
COG0016	0.72347	COG1121	1.13051	COG1014	1.04632	COG5257	0.87199
COG0013	0.91729	COG0456	1.05452	COG0836	1.15020	COG0855	1.18751
COG0098	0.56956	COG0365	0.77654	COG0839	1.03830	COG1559	1.48917
COG0343	0.78057	COG0111	0.87715	COG0542	0.89556	COG2520	1.62905
COG0750	1.39272	COG0249	1.17856	COG0228	0.59366	COG1685	1.22133
COG0097	1.00246	COG4974	1.45818	COG1573	1.05012	COG2082	0.82301
COG0092	0.64926	COG0206	0.54019	COG0816	1.12410	COG2509	0.84302
COG0244	1.05110	COG1009	0.83301	COG0566	1.33356	COG0569	1.15431
COG0532	0.72680	COG0289	0.95033	COG0560	1.03877	COG0601	1.20093
COG0231	0.69596	COG0484	0.91040	COG1235	1.04185	COG1727	0.86057
COG0081	0.80031	COG0007	0.79837	COG1537	1.55506	COG0623	0.80780
COG0087	0.76958	COG1257	0.71650	COG1709	1.02474	COG1252	1.32188
COG0197	0.61709	COG0402	1.20095	COG1207	1.09790	COG1116	0.87597
COG2812	1.11201	COG1158	0.58018	COG2139	0.73993	COG0662	0.99834
COG0049	0.55139	COG1156	0.52007	COG1912	1.32528	COG0661	1.27160
COG0250	0.79122	COG2217	1.05886	COG0404	0.95515	COG1339	1.52523
COG0611	1.27552	COG0445	0.77927	COG0669	0.79079	COG0680	1.13726
COG0174	0.78501	COG1782	0.82351	COG1136	0.66862	COG0446	1.37251
COG0341	1.09573	COG0377	0.50361	COG2239	1.36952	COG0160	0.87754
COG0528	1.16007	COG1514	1.58883	COG0168	1.33363	COG0166	1.31055
COG0099	0.68518	COG0129	0.75540	COG0318	1.15754	COG1370	1.74534
COG0143	0.97782	COG0330	0.92910	COG0319	1.11615	COG0768	1.47845
COG0449	0.97982	COG1058	1.15933	COG1193	1.60938	COG1325	1.67022
COG0020	1.24637	COG0041	0.56519	COG1192	1.23492	COG1491	1.00016
COG0495	0.88084	COG0825	0.88937	COG0769	1.23518	COG0375	1.49351
COG2262	1.09423	COG0009	0.99503	COG0766	0.97067	COG0591	1.24755
COG0151	0.90947	COG0613	0.78929	COG0379	0.89877	COG0743	0.85781
COG0522	0.66373	COG1249	1.14267	COG0590	0.97253	COG1730	1.77445
COG0018	1.26960	COG0517	0.78442	COG0123	1.02003	COG0703	1.87040
COG0441	0.99291	COG0512	0.72607	COG1650	1.74272	COG1097	1.04985
COG0126	1.08415	COG0157	0.96035	COG0720	0.92977	COG1679	1.16357
COG0358	0.64552	COG1793	0.91862	COG0728	1.92991	COG2016	1.42142
COG0060	0.82364	COG1364	0.88986	COG0335	0.57289	COG0331	1.13728
COG0498	0.75476	COG0131	0.77273	COG0332	1.07638	COG1849	1.25738
COG0008	0.71071	COG0713	0.74708	COG1841	0.58139	COG4172	0.95584
COG0037	0.89751	COG0297	1.11054	COG1460	1.36887	COG1281	1.34124
COG1293	1.58192	COG0059	0.61443	COG0253	0.98016	COG1079	1.72025
COG0052	0.70071	COG0501	0.65141	COG0252	0.97991	COG1056	0.74613

COG0541	0.91729	COG0520	0.94361	COG2038	0.92022	COG1586	0.65624
COG0462	0.98914	COG0209	0.72251	COG1829	1.62293	COG1631	0.63565
COG0100	0.32215	COG1219	0.55214	COG0068	1.17027	COG0864	0.77593
COG0048	0.45755	COG0605	0.80736	COG0275	1.05488	COG1595	0.98005
COG0452	1.09789	COG0420	1.08108	COG1051	0.87224	COG0021	1.03339
COG0030	1.55036	COG2255	0.81615	COG0515	1.30067	COG0026	1.16071
COG0072	1.14843	COG0461	1.18150	COG1030	1.45098	COG0821	0.69381
COG0505	0.79836	COG0469	1.17664	COG0023	0.41005	COG0004	0.98804
COG0423	0.75524	COG0681	1.11063	COG0234	0.60813	COG1980	0.60786
COG0102	0.88018	COG1488	1.05865	COG1205	1.24680	COG1691	1.09648
COG0064	0.86711	COG0323	1.23547	COG0807	1.13263	COG1226	1.04001
COG0061	1.44143	COG0761	0.94939	COG3642	1.10257	COG0638	0.71963
COG0539	0.85657	COG0372	0.72966	COG1522	0.83708	COG0389	1.25994
COG0552	0.70265	COG1358	0.54296	COG1527	1.99849	COG0652	0.51199
COG0200	0.66051	COG0740	0.67582	COG1389	1.07081	COG0651	1.39146
COG0015	0.92994	COG0745	1.10459	COG1385	1.34786	COG1387	1.17371
COG0644	0.93176	COG0746	1.47474	COG2263	2.74794	COG1384	1.21352
COG1196	1.47375	COG0721	1.14903	COG0438	1.61094	COG1900	1.26485
COG0373	1.31021	COG1093	1.16562	COG0673	1.22747	COG1798	1.35330
COG0046	1.18628	COG1077	0.58895	COG2243	1.30364	COG1303	1.21823
COG0237	1.30718	COG0066	0.86123	COG2241	1.53438	COG1300	1.62818
COG0177	1.12825	COG0182	1.09469	COG0479	0.87784	COG0799	0.75827
COG0119	0.73486	COG0045	0.84648	COG1126	0.77581	COG0170	1.63807
COG0608	1.42897	COG1044	1.03873	COG1437	0.94515	COG1968	1.56306
COG0621	1.20230	COG0210	0.94136	COG0309	1.16254	COG0367	1.41626
COG0460	0.90684	COG0618	0.82369	COG0307	1.17850	COG1515	1.50605
COG0106	0.98183	COG0576	1.02905	COG1308	0.78767	COG0772	1.62318
COG0150	1.03973	COG1222	0.70630	COG1163	1.15282	COG1646	1.06186
COG0024	0.89003	COG0470	1.63869	COG0014	0.87291	COG1645	1.30796
COG0496	1.14687	COG1498	1.08868	COG0793	1.07617	COG3252	0.90987
COG0574	0.80595	COG1499	1.63816	COG2433	1.78802	COG0283	1.06986
COG0088	1.03173	COG1492	1.10080	COG0360	1.05611	COG1668	1.47739
COG2226	1.04574	COG0303	1.40653	COG1180	0.88611	COG2061	1.39521
COG0329	0.89497	COG0171	1.17206	COG0777	0.74292	COG1412	1.01441
COG0152	0.95491	COG0139	0.59543	COG1648	1.43158	COG1413	2.03424
COG0342	1.19611	COG1087	1.04984	COG0347	0.58525	COG0730	1.72258
COG0137	0.93423	COG0074	0.57353	COG2064	1.09163	COG0731	1.19713
COG0195	0.99073	COG0294	1.17745	COG1872	1.39609	COG1473	1.24622
COG0548	0.88610	COG1047	1.32694	COG1067	0.75886	COG2108	1.38058
COG0149	0.68745	COG1624	1.05126	COG1599	1.57175	COG0718	1.03812
COG0127	0.90122	COG0203	0.66105	COG1040	1.26847	COG1043	1.30348
COG0181	0.92494	COG0010	0.73317	COG1831	1.55949	COG1622	1.05466
COG0044	1.16396	COG0544	1.43108	COG0056	0.65005	COG1628	1.99996
COG0188	0.82835	COG0838	0.93030	COG1459	1.33214	COG1830	0.64211
COG0080	0.55043	COG0547	1.15402	COG0263	0.94948	COG0055	0.44789
COG0084	1.35565	COG0019	1.15644	COG0261	0.97888	COG1452	1.71589
COG0459	0.76643	COG1746	1.48106	COG0196	1.26201	COG1008	1.11611
COG0130	0.87954	COG0602	1.37891	COG0524	1.15785	COG0546	1.63506
COG0078	0.87954	COG1208	1.22686	COG0543	1.16311	COG1763	0.91157
COG0054	0.78785	COG0422	0.47169	COG0223	1.16463	COG1995	1.48861
COG0012	0.98610	COG0648	1.15306	COG0221	0.96906	COG1992	1.57132
COG2256	1.09824	COG3425	0.69307	COG1571	1.63061	COG0220	1.27543
COG0094	0.94158	COG0616	1.10984	COG0482	1.25094	COG0227	0.82164
COG0090	0.68000	COG0442	1.01943	COG0815	1.86908	COG0224	0.81896
COG0165	1.13653	COG0317	1.05641	COG0817	1.17767	COG1518	1.27173
COG0103	0.51619	COG1355	1.10836	COG0563	1.18852	COG1721	1.07445

COG0104	0.93108	COG0299	0.89718	COG0607	1.02145	COG1534	1.31853
COG0215	0.75964	COG0747	1.36849	COG0609	1.29973	COG1899	1.04362
COG2890	1.63654	COG0357	1.16865	COG0628	1.40409	COG0643	0.99586
COG0002	1.02139	COG1676	1.36212	COG0626	0.94278	COG0095	1.50039
COG0794	0.71903	COG1467	1.36572	COG0620	1.05048	COG0407	1.18716
COG1185	1.01255	COG4775	1.66210	COG1390	1.49560	COG0405	1.35045
COG0113	0.81852	COG0183	0.73762	COG3635	1.26950	COG1132	1.05303
COG0051	0.35033	COG0247	0.72174	COG1259	0.85034	COG1938	1.39553
COG0017	0.79976	COG0212	1.24342	COG0399	0.90712	COG1933	1.17167
COG0606	1.06948	COG0493	0.88038	COG0665	1.67869	COG0467	0.45477
COG0649	0.67223	COG0005	0.65929	COG0668	0.87568	COG0687	1.31743
COG0091	0.62947	COG1530	0.97552	COG1137	0.68666	COG1331	1.41991
COG0465	0.69651	COG0809	1.21114	COG0463	0.85738	COG1216	1.37655
COG1197	1.07689	COG0571	1.46134	COG1210	1.10644	COG1311	1.26972
COG4770	0.62865	COG0413	0.89587	COG0444	0.91834	COG1171	0.76063
COG0185	0.48139	COG1102	1.21385	COG1173	1.18957	COG0781	0.81548
COG0001	0.80702	COG2101	0.45266	COG2078	0.95997	COG0162	1.13176
COG0558	1.15246	COG2220	0.97497	COG1867	1.50495	COG3264	1.24604
COG0519	0.72821	COG0450	0.45990	COG0352	1.13434	COG1324	1.04268
COG0472	1.31439	COG1161	1.28711	COG0350	0.78654	COG2058	0.69646
COG1160	0.97641	COG0179	0.79900	COG0355	0.92744	COG0378	0.75317
COG0368	0.98358	COG1363	0.99962	COG1446	1.00630	COG0596	1.45504
COG0034	0.98243	COG0589	1.24388	COG1634	1.46318	COG0598	2.08279
COG0136	0.67706	COG2608	1.19206	COG0242	0.96408	COG0725	1.49758
COG0028	0.81815	COG0735	0.99656	COG1012	1.06214	COG1092	1.21653
COG0079	0.94607	COG1007	1.58196	COG1011	1.30597	COG0707	1.38952
COG0057	0.79112	COG1474	0.93944	COG1204	1.16127	COG0251	0.90564
COG0141	0.85745	COG0714	0.88056	COG0614	1.25783	COG1071	0.93362
COG0455	0.63468	COG0292	0.71503	COG1697	0.77129	COG1610	0.98618
COG0312	1.16558	COG1028	0.72986	COG0802	1.30083	COG1825	1.25711
COG0128	1.01217	COG0193	1.10100	COG2511	1.02827	COG0069	0.72027
COG0256	0.78412	COG0194	1.09254	COG0189	1.07355	COG1449	1.11889
COG0142	0.90206	COG1812	0.88485	COG1717	1.01103	COG1055	1.32442
COG0704	1.22595	COG0039	0.88071	COG0381	1.34981	COG0514	1.13109
COG0006	1.07255	COG1994	1.20078	COG0432	1.08837	COG0245	0.91995
COG1241	1.05563	COG0564	1.11510	COG0430	1.07220	COG1033	1.59165
COG1109	0.89082	COG1395	1.42286	COG1526	1.49127	COG5016	0.85651
COG0082	0.65596	COG1209	1.28124	COG1386	1.18034	COG1019	1.49034
COG4992	1.07922	COG0685	1.79410	COG1382	0.72594	COG2092	0.75181
COG1122	1.17534	COG1155	0.72354	COG2264	1.42919	COG2095	1.43483
COG0458	0.75390	COG1154	0.97881	COG2265	1.25692	COG0824	1.14305
COG0396	0.98539	COG1500	0.69556	COG2109	1.14212	COG0806	1.62414
COG0172	0.90678	COG2151	0.69136	COG1909	1.32172	COG2518	1.00022
COG0587	1.08299	COG1976	1.45881	COG0478	1.23113	COG0508	0.90555
COG1080	0.91088	COG0311	1.01857	COG1144	0.75223	COG0507	1.07365
COG0527	0.87136	COG1198	1.33928	COG1940	1.03942	COG1889	0.86414
COG0557	1.08670	COG0353	0.93423	COG1948	1.52585	COG1711	1.18547
COG1890	1.08465	COG1675	1.31539	COG0308	1.48162	COG3383	1.06813
COG0096	0.69294	COG1469	0.99360	COG1162	1.30837	COG0411	0.76793
COG0147	0.84997	COG0706	1.06714	COG3118	0.63144	COG0679	1.67907
COG0167	0.95441	COG1078	0.99323	COG0776	0.56330	COG2102	1.13618
COG0169	0.98332	COG1611	1.30807	COG2041	1.31257	COG1907	1.39847
COG0108	0.84084	COG0047	0.89445	COG1436	1.30917	COG0475	1.45187
COG0107	0.65400	COG0216	0.81089	COG0751	1.76135	COG0326	1.15121
COG0595	0.95633	COG0218	1.17666	COG0281	0.82359	COG1496	1.40852
COG0124	0.97008	COG0533	0.86977	COG0285	1.12061	COG1497	1.80597

COG0180	1.01417	COG1013	0.74062	COG0135	0.93486	COG2147	0.82323
COG0499	0.60978	COG0497	1.26682	COG1084	1.21556	COG2141	1.12313
COG2875	1.16693	COG2097	0.94983	COG1066	0.84975	COG0155	1.02946
COG0516	0.70471	COG0820	0.96876	COG1607	0.95024	COG0302	0.98926
COG0476	0.92816	COG2519	1.51247	COG1245	0.97805	COG0306	1.60423
COG0154	0.69224	COG0632	1.05555	COG2004	1.36238	COG0796	1.20696
COG1304	1.16936	COG0639	0.98137	COG0076	1.63156	COG0036	0.74242
COG0361	0.43316	COG0433	0.68741	COG0070	1.04339	COG1369	1.97057
COG0118	1.06187	COG2125	0.85881	COG1290	0.59848	COG1181	0.97375
COG0112	0.94200	COG1702	0.85241	COG0711	1.21626	COG0583	1.17626
COG0719	1.00605	COG0265	0.94958	COG1838	0.75951	COG0581	1.51238
COG0717	0.84552	COG0697	2.16941	COG0050	0.44279	COG1644	0.52858
COG0503	0.82158	COG0451	0.80314	COG0053	1.44868	COG1422	1.69756
COG0192	0.55936	COG1797	1.14790	COG1024	0.92053	COG0344	1.67371
COG0198	0.55897	COG1792	1.24772	COG0858	0.91173	COG0340	1.21850
COG0202	0.69593	COG5002	1.10840	COG1003	0.76518	COG1439	1.46642
COG1005	1.01363	COG0774	1.34896	COG1767	1.68024	COG1343	1.39888
COG0483	1.30433	COG0115	0.98237	COG1997	0.60952	COG0752	0.82190
COG0481	0.66454	COG1641	1.48532	COG0226	0.98448	COG1082	0.92789
COG0568	0.44283	COG0035	0.85042	COG1570	1.47224	COG0534	1.81292
COG0556	0.79318	COG0419	1.39131	COG0811	0.74025	COG1061	1.19285
COG1394	1.22875	COG1475	1.01311	COG0812	1.16576	COG0538	0.49461
COG0148	0.81208	COG1041	1.53101	COG0624	1.11829	COG1606	1.24210
COG0443	0.52347	COG1340	0.63471	COG1258	1.51707	COG1472	1.33457
COG0322	0.95167	COG0264	0.96710	COG0640	0.56009	COG0293	1.25130
COG0371	1.17772	COG5256	0.50613	COG2138	0.92928	COG1832	1.11229
COG1354	1.28555	COG1004	0.87420	COG2236	1.22966	COG0502	1.11265
COG0125	1.53213	COG1545	1.03234	COG0682	1.30004	COG0266	1.55102
COG0065	0.73328	COG4591	1.38432	COG0683	1.44488	COG0268	1.06097
COG1503	0.79035	COG0603	1.10170	COG1951	1.11886	COG0038	1.18332
COG0187	0.69890	COG0551	1.08648	COG1959	1.22895	COG0204	1.37698
COG0184	0.50182	COG0424	1.03253	COG0327	1.39259	COG1547	1.40147
COG0040	0.99863	COG1117	0.78401	COG0782	0.84838	COG2524	1.15283
COG0492	0.82274	COG0663	1.04818	COG1781	0.79345	COG1577	1.13756
COG1718	1.32372	COG1131	0.74368	COG0313	1.14655	COG2085	1.37927
COG0653	0.80515	COG2237	1.68585	COG1371	0.92446	COG0818	1.58719
COG0436	0.84159	COG0466	0.74537	COG0763	1.79110	COG1741	1.29836
COG0305	0.93466	COG1159	1.18971	COG1327	1.13020	COG0553	1.47186
COG0178	0.73279	COG0144	1.35009	COG1321	0.83275	COG0629	0.98506
COG0585	1.60715	COG1217	0.87300	COG0298	0.70360	COG2271	1.56800
COG0284	1.25229	COG0787	1.48488	COG1736	1.21198	COG1250	0.72322
COG0134	1.10775	COG0324	1.09278	COG1094	0.98090	COG1253	1.96277
COG1601	0.71133	COG0320	0.80097	COG0336	0.92780	COG1911	0.75784
COG0077	1.03101	COG0315	1.04253	COG0270	0.91029	COG0409	0.68974
COG0075	1.01752	COG1199	1.26756	COG0277	0.91609	COG2804	1.12935
COG1836	1.29462	COG1195	1.54378	COG0240	1.26614	COG1931	1.06532
COG0540	1.03167	COG0109	1.22645	COG0243	1.31738	COG1936	1.90669
COG1236	1.17371	COG0359	1.07742	COG1045	0.91055	COG0464	0.74972
COG1234	0.98608	COG1405	0.33035	COG0217	0.81506	COG2219	1.44477
COG0689	0.55839	COG1095	0.80195	COG1032	0.96888	COG1211	1.18723
COG1157	0.52004	COG1674	1.02340	COG0842	1.86255	COG1958	0.41729
COG0440	0.60088	COG0334	0.90623	COG0022	0.68711	COG0325	1.14396
COG0163	0.99505	COG0337	1.07665	COG1779	1.43505	COG1194	0.95444
COG0164	0.95379	COG1466	1.90513	COG0235	1.18436	COG0370	1.15665
COG0314	1.30155	COG0272	1.15644	COG1010	0.97342	COG1351	0.44773
COG0101	1.16739	COG1632	0.71340	COG0494	1.13153	COG0122	1.11726

COG0592	1.48355	COG1215	1.35176	COG1201	1.13673	COG0254	0.52810
COG0593	1.19911	COG0233	0.94591	COG1243	0.95016	COG1862	0.90070
COG0258	0.95117	COG0491	1.45490	COG0617	1.18132	COG1409	0.80097
COG0062	1.21055	COG1200	1.07820	COG0619	1.41570	COG0726	0.83126
COG0186	0.58232	COG0805	1.74262	COG0803	1.41576	COG1096	1.81187
COG0029	0.86727	COG1225	0.89842	COG0575	1.20438	COG1098	1.19561
COG2896	0.96879	COG0509	0.95793	COG1508	1.35495	COG2019	0.85187
COG1985	1.09801	COG0388	1.25387	COG3839	0.55353	COG1845	1.14848
COG0615	0.82511	COG2872	0.97568	COG2123	1.03901	COG1287	1.36792
COG0083	1.27694	COG1381	1.62398	COG0416	1.23690	COG1073	1.01576
COG0304	0.91093	COG0417	0.96819	COG0677	1.20971	COG0274	0.99242
COG0173	0.76868	COG0674	0.89949	COG1104	0.93601	COG1581	0.45232
COG1187	1.38366	COG2814	1.69719	COG0511	0.65440	COG0213	0.88215
COG0133	0.67322	COG1108	1.07478	COG0474	1.19557	COG0843	0.86461
COG1855	1.23694	COG0513	0.85539	COG0691	0.81610	COG0841	1.38130
COG0071	0.94871	COG1903	1.11166	COG0328	0.92432	COG0849	1.48285
COG0521	1.27099	COG1120	1.60782	COG0457	2.12552	COG4651	1.46416
COG0222	0.79697	COG1167	1.26835	COG1328	0.88951	COG0535	0.91316
COG0486	1.01756	COG1183	0.45413	COG0391	0.86541	COG5011	1.40574
COG0565	1.60466	COG0771	1.38120	COG1947	1.60926	COG0239	1.04314
COG0561	1.63056	COG0773	1.05234	COG0301	1.02523	COG1549	1.65402
COG1270	1.42894	COG0345	1.01348	COG0795	1.67390	COG0555	1.23044
COG1190	0.89331	COG1432	0.28719	COG0031	1.02838	COG1539	1.41428
COG0105	0.64453	COG0287	1.14097	COG1189	1.44390	COG1561	1.45297
COG0120	1.06911	COG0138	0.87677	COG0770	1.12178	COG0573	1.31412
COG0159	1.13529	COG0536	0.97498	COG0116	1.37324	COG0631	0.87550
COG1587	1.60049	COG1060	1.31261	COG0758	1.26108	COG0637	1.46259
COG0042	1.14244	COG1600	1.17508	COG1088	0.71513		
