# Supplementary figures and images for: Universal Pacemaker of Genome Evolution
Source: PLoS Comput Biol. 2012 Nov 29;8(11):e1002785. doi: 10.1371/journal.pcbi.1002785 (PMC3510094; doi:10.1371/journal.pcbi.1002785)

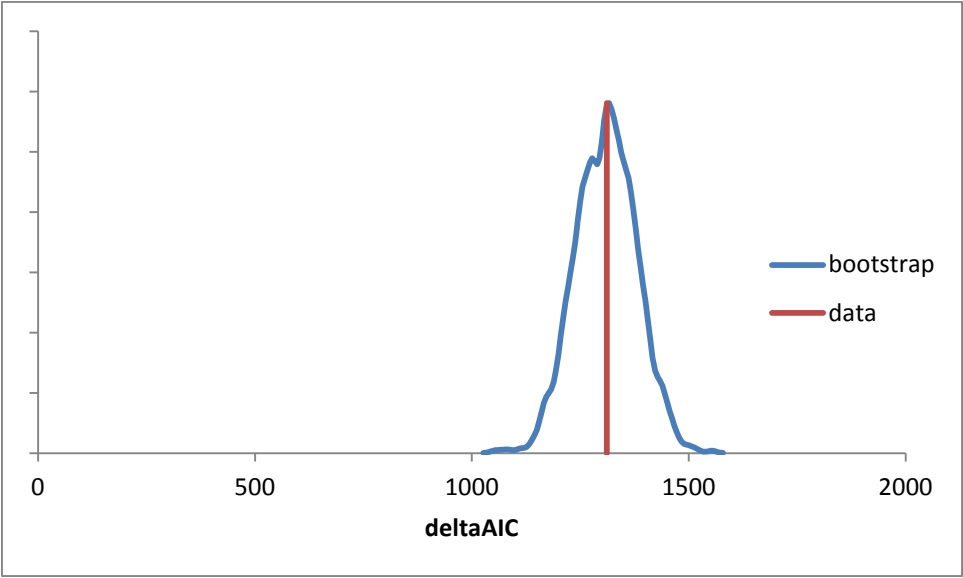

Supplement: Figure S2 — Distribution of the ΔAIC values for 1000 bootstrap samples (the curve was obtained by Gaussian-kernel smoothing of the individual data points). The red line indicates the ΔAIC value for the original set of GTs (1310.8). (PDF) [file pcbi.1002785.s002.pdf]

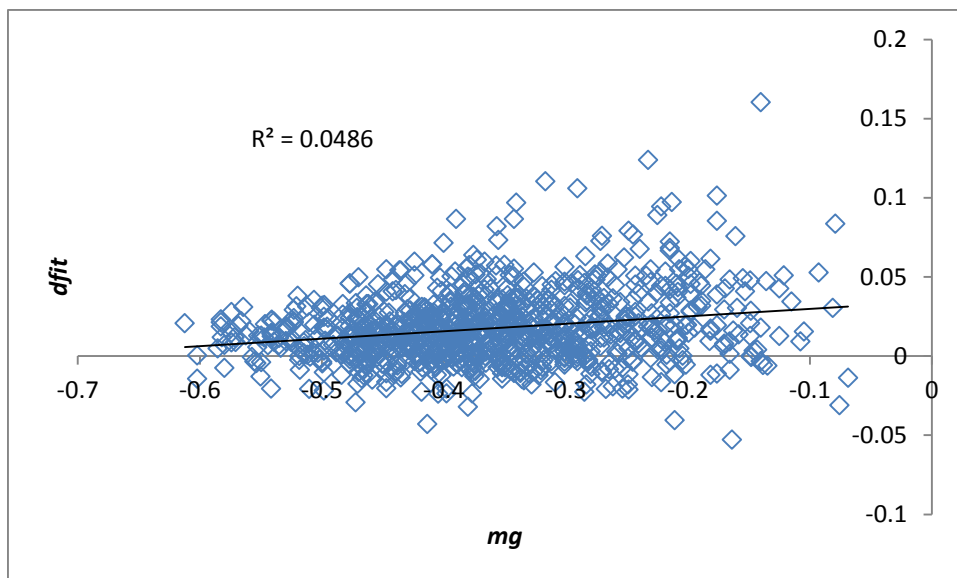

A

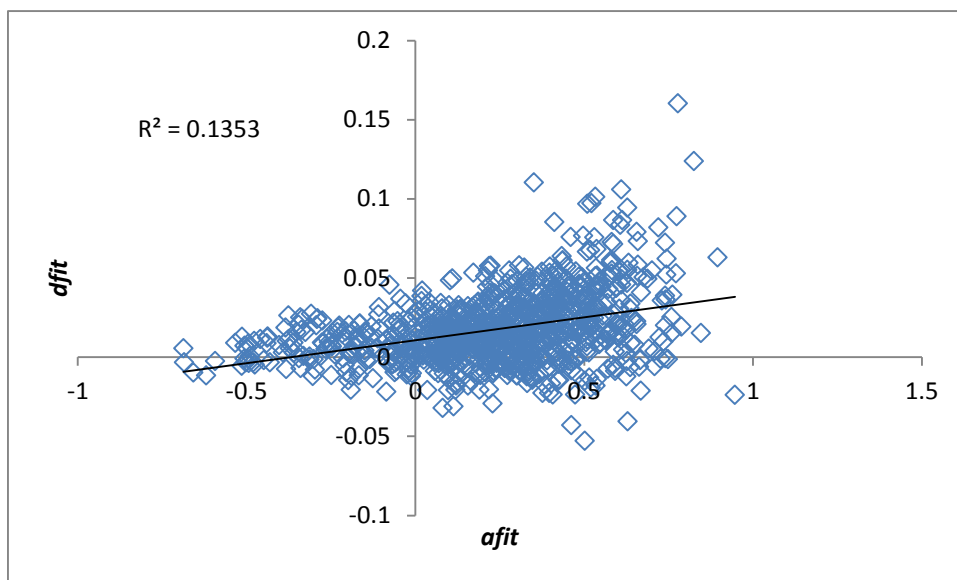

B

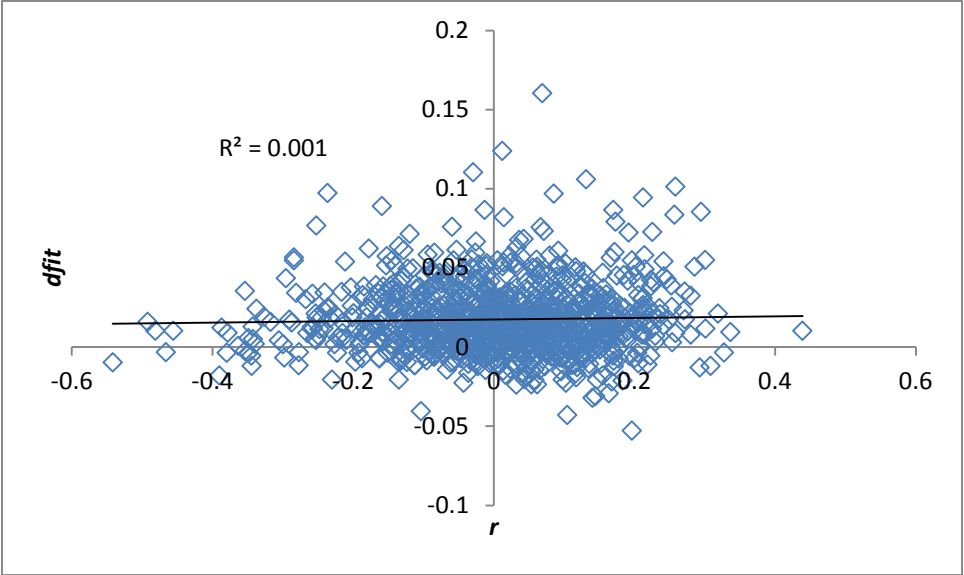

C

Supplement: Figure S3 — A: Relative goodness of fit for the UPM vs the MC model (dfit) plotted against the fraction of original GT leaves retained in MAST (mg). B: Relative goodness of fit for the UPM vs the MC model (dfit) plotted against the average goodness of fit (afit). C: Relative goodness of fit for the UPM vs the MC model (dfit) plotted against the relative evolution rate (r).C. (PDF) [file pcbi.1002785.s003.pdf]
