# Supplementary material for: Universal Pacemaker of Genome Evolution
Source: PLoS Comput Biol. 2012 Nov 29;8(11):e1002785. doi: 10.1371/journal.pcbi.1002785 (PMC3510094; doi:10.1371/journal.pcbi.1002785)
Supplement: Text S2 — Maximum Likelihood estimates for the supertree edge lengths and gene evolution rates. (DOCX) [file pcbi.1002785.s005.docx]

**Maximum Likelihood estimate for the supertree edge lengths and gene evolution rates**

Consider a rooted supertree (ST) with a fixed topology. The ST encompasses a set of edges ***e*** defined by the ST topology and a set of unknown edge lengths ***t***. Consider a set of unrooted GTs reduced to MAST with the given ST. Each GT encompasses a set of edges with known edge lengths and an unknown gene-specific evolution rate (***b****_k_*, ***l****_k_* and *r_k_* for the *k*-th GT, respectively). Each edge of each GT uniquely maps to an ST path ***e****_j_*, that is a subset of adjacent edges in the ST (*b_k,i_* ≡ ***e****_j_* where ***e****_j_* ⊆ ***e*** for the *i*-th edge of the *k*-th GT).

Let $t_{j}=\sum_{x\in\boldsymbol{e}_{\boldsymbol{j}}} t_{x}$ be the length of the path ***e****_j_*. We assume that the length of the *i*-th edge of the *k*-th GT is related to the length of the corresponding ST path ***e****_j_*:

$$l_{i,k}=t_{j}r_{k}\varepsilon_{i,k}$$

where ε*_i,k_* is the multiplicative deviation factor for the given edge. We further assume that the deviation is random, independent for branches both within and between GTs, and comes from a lognormal distribution with the mean of 1 and an arbitrary variance, translating to a model with an additive normally distributed deviation in the logarithmic scale (i.e. ln ε*_i,k_* ~ *N*(0,σ^2^)).

Given ***t*** and ***r***, the expectation for the logarithm of the length of the *i*-th edge of the *k*-th GT is:

$$\mu_{i,k}=\left\langle\ln l_{i,k} \right\rangle=\left\langle\ln t_{j} \right\rangle+\left\langle\ln r_{k} \right\rangle+\left\langle\ln\varepsilon_{i,k} \right\rangle=\ln t_{j}+\ln r_{k}$$

and the likelihood of observing the length *l_i,k_* is:

$$\Pr\left\{ l_{i,k} | \boldsymbol{t},\boldsymbol{r} \right\}=\frac{1}{\sigma\sqrt{2\pi}}\exp\left( -\frac{\left( \ln l_{i,k}-\mu_{i,k} \right)^{2}}{2\sigma} \right)=\frac{1}{\sigma\sqrt{2\pi}}\exp\left( -\frac{\left( \ln l_{i,k}-\ln t_{j}-\ln r_{k} \right)^{2}}{2\sigma} \right)=\frac{1}{\sigma\sqrt{2\pi}}\exp\left( -\frac{E_{i,k}^{2}}{2\sigma} \right)$$

where *E*^2^*_i,k_* = (ln *l_i,k_* ‑ ln *t_j_* ‑ ln *r_k_*)^2^. For all observed edge lengths in all GTs (***l***), the likelihood function is

$$L(\boldsymbol{l}|\boldsymbol{t},\boldsymbol{r})=\prod_{k} \prod_{i} \Pr\left\{ l_{i,k} | \boldsymbol{t},\boldsymbol{r} \right\}$$

In the logarithmic scale:

$$\ln L\left( \boldsymbol{l} | \boldsymbol{t},\boldsymbol{r} \right)=\sum_{k} \sum_{i} \ln\frac{1}{\sigma\sqrt{2\pi}}\exp\left( -\frac{E_{i,k}^{2}}{2\sigma} \right)=-\frac{n}{2}\ln\sigma^{2}-\frac{n}{2}\ln2\pi-\frac{\sum_{k} \sum_{i} E_{i,k}^{2}}{2\sigma^{2}}$$

where *n* is the total number of GT edges ($n=\sum_{k} \sum_{i} 1$). Designating the residual sum of squares $E^{2}=\sum_{k} \sum_{i} E_{i,k}^{2}$ and substituting the estimate for σ^2^

$$\hat{\sigma^{2}}=\frac{E^{2}}{n-1}\approx\frac{E^{2}}{n}$$

for large *n*, we obtain:

$$\ln L(\boldsymbol{l}|\boldsymbol{t},\boldsymbol{r})\approx-\frac{n}{2}\ln\frac{E^{2}}{n}-\frac{n}{2}\ln2\pi-\frac{n}{2}$$

Because *n* is constant for a given data set, finding the maximum of *L*(***l*** | ***t***,***r***) is equivalent to finding the minimum of *E*^2^.

**Optimization procedure**

Least Squares (LS) is called linear if the residuals are linear for all unknowns. Linear LS can be represented in a matrix format which has a closed form solution (given that the columns of the matrix are linearly independent). However, our formulation requires taking logs over sums of unknowns in the case where a GT edge corresponds to a path in ST ($\ln t_{j}=\ln\sum_{x\in\boldsymbol{e}_{\boldsymbol{j}}} t_{x}$). Then, the problem becomes non-linear with respect LS and can be solved only using numerical algorithms where the solution is obtained by iteratively refining the parameter values. This approach requires supplying initial values for the parameters. The goodness of the initial value estimation is critical for the convergence time of the iterative method and the risk of being trapped in local maximum points. We employed the following strategy for determining the initial values: For each ST edge, we computed the mean value of the sum over all GT edges that uniquely correspond to the given edge. The latter also creates a ranking between the gene rates (even when edges are missing). Therefore, if we assign one gene a specific rate value (e.g. the length of some edge), we obtain initial rate values for all genes. It can be easily shown that, if there are no errors in rates (i.e. σ^2^ = 0), the above procedure yields the accurate (ML) values for all unknowns.

To perform the LS optimization, we used the function *fmin_slsqp()* that is part of the *scipy.optimize* package of Python which minimizes a function using sequential least squares programming. The function admits also a set of constraints that are necessary for our calculation. In both the MC and the UPM models, both the ST edges and the GT rates were constrained to positive values. For the UPM model, the distances from a node to any leaf in a subtree under that node were set equal for all subtrees under that node. It can be shown by induction that this constraint implies an ultrametric tree. Thus, we have a constraint for every internal node; in a rooted binary tree with *m* leaves, there are *m* ‑ 1 such nodes.
