# Supplementary material for: Universal Pacemaker of Genome Evolution
Source: PLoS Comput Biol. 2012 Nov 29;8(11):e1002785. doi: 10.1371/journal.pcbi.1002785 (PMC3510094; doi:10.1371/journal.pcbi.1002785)
Supplement: Text S3 — Goodness of fit for the MC and UPM models: bootstrap analysis and dependence on evolutionary and functional charateristics of gene families. (DOCX) [file pcbi.1002785.s006.docx]

**Bootstrap analysis**

The sets of 967 pairs of *E*^2^*_MC,k_* and *E*^2^*_UPM,k_* values for each GT were sampled with replacement 967 times. The Δ*AIC* values were computed using the sums of the sampled values. This procedure was repeated 1000 times.The range of the Δ*AIC* values in the bootstrap sample is [1051.9, 1553.3]; 95% of the samples fell within the [1172.0, 1443.3] interval. (see Supporting Figure S2)

**Goodness of fit**

Analysis of the dependence of goodness of fit on other variables was performed using R as follows:

dat <- read.table("tmp.dat",header=TRUE,sep="\t")

Original data are transformed to a log scale:

dat$m <- log(dat$NM) # MAST GT size

dat$g <- log(dat$NG) # original GT size

dat$r <- log(dat$rUPM)

dat$cn <- log(dat$e2MC) - log(2*dat$NM-3)

dat$pn <- log(dat$e2UPM) - log(2*dat$NM-3)

dat$afit <- -(dat$cn+dat$pn)/2 # average MC,UPM fit

# per MAST edge

dat$dfit <- log(dat$e2MC) - log(dat$e2UPM) # difference between

# UPM and MC fit

dat$mg <- dat$m - dat$g # fraction of GT leaves

# retained in MAST

attach(dat)

We were primarily interested in the trees that showed a better fit to UPM than to MC. Thus, we apply a linear model to explain the *dfit* value:

m00 <- lm(dfit ~ m+g+r+afit)

m01 <- step(m00)

summary(m01)

Call:

lm(formula = dfit ~ m + g + r + afit)

Residuals:

Min 1Q Median 3Q Max

-0.1854498 -0.0265786 0.0001418 0.0256124 0.2836366

Coefficients:

Estimate Std. Error t value Pr(>|t|)

(Intercept) 0.025600 0.024122 1.061 0.288850

m 0.033381 0.009398 3.552 0.000401 ***

g -0.026620 0.006554 -4.062 5.27e-05 ***

r -0.011719 0.005304 -2.210 0.027368 *

afit 0.027681 0.002643 10.474 < 2e-16 ***

---

Signif. codes: 0 '***' 0.001 '**' 0.01 '*' 0.05 '.' 0.1 ' ' 1

Residual standard error: 0.04584 on 962 degrees of freedom

Multiple R-squared: 0.1583, Adjusted R-squared: 0.1548

F-statistic: 45.24 on 4 and 962 DF, p-value: < 2.2e-16

We find that all variables significantly affect *dfit*. However, *m* (MAST GT size) and *g* (original GT size) have coefficients with opposite signs and similar absolute values. We further tested the hypothesis that it is the fraction of original GT leaves retained in MAST (*mg*) that is important:

m10 <- lm(dfit ~ mg+g+r+afit)

m11 <- step(m10)

summary(m11)

Call:

lm(formula = dfit ~ mg + r + afit)

Residuals:

Min 1Q Median 3Q Max

-0.1859028 -0.0266123 0.0004592 0.0256792 0.2812774

Coefficients:

Estimate Std. Error t value Pr(>|t|)

(Intercept) 0.047747 0.006156 7.757 2.21e-14 ***

mg 0.026975 0.006543 4.123 4.07e-05 ***

r -0.013612 0.004915 -2.770 0.00572 **

afit 0.028357 0.002545 11.141 < 2e-16 ***

---

Signif. codes: 0 '***' 0.001 '**' 0.01 '*' 0.05 '.' 0.1 ' ' 1

Residual standard error: 0.04583 on 963 degrees of freedom

Multiple R-squared: 0.1575, Adjusted R-squared: 0.1549

F-statistic: 60.03 on 3 and 963 DF, p-value: < 2.2e-16

anova(m01,m11)

Analysis of Variance Table

Model 1: dfit ~ m + g + r + afit

Model 2: dfit ~ mg + r + afit

Res.Df RSS Df Sum of Sq F Pr(>F)

1 962 2.0212

2 963 2.0231 -1 -0.0018944 0.9017 0.3426

In the presence of *mg*, *g* is excluded from the model without loss of significance (ANOVA test p-value of 0.34). Thus, we conclude that the trees that fit UMP better than MC tend to:

- retain higher fraction of the original GT leaves in MAST regardless of the total number of species in the tree (these are the trees that are least affected by HGT and tree reconstruction artifacts)
- show a better fit to ST (that trees that are least dispersed)
- are characterized by low evolution rate

Finally, to determine whether functional characteristics of the gene family play a role (*Class* variable), we used the following procedure:

r20 <- lm(dfit ~ mg+r+afit+Class)

r21 <- step(r20)

summary(r21)

Call:

lm(formula = dfit ~ mg + r + afit)

Residuals:

Min 1Q Median 3Q Max

-0.1859028 -0.0266123 0.0004592 0.0256792 0.2812774

Coefficients:

Estimate Std. Error t value Pr(>|t|)

(Intercept) 0.047747 0.006156 7.757 2.21e-14 ***

mg 0.026975 0.006543 4.123 4.07e-05 ***

r -0.013612 0.004915 -2.770 0.00572 **

afit 0.028357 0.002545 11.141 < 2e-16 ***

---

Signif. codes: 0 '***' 0.001 '**' 0.01 '*' 0.05 '.' 0.1 ' ' 1

Residual standard error: 0.04583 on 963 degrees of freedom

Multiple R-squared: 0.1575, Adjusted R-squared: 0.1549

F-statistic: 60.03 on 3 and 963 DF, p-value: < 2.2e-16

The *Class* variable was excluded from the model in the course of the stepwise reduction. Consequently, we find that the functional assignment of the gene family is unimportant.

The results of this analysis are graphically presented in the Supporting Figure S3.
